# A Typical Immune T/B Subset Profile Characterizes Bicuspid Aortic Valve: In an Old Status?

**DOI:** 10.1155/2018/5879281

**Published:** 2018-04-05

**Authors:** Carmela R. Balistreri, Silvio Buffa, Alberto Allegra, Calogera Pisano, Giovanni Ruvolo, Giuseppina Colonna-Romano, Domenico Lio, Giuseppe Mazzesi, Sonia Schiavon, Ernesto Greco, Silvia Palmerio, Sebastiano Sciarretta, Elena Cavarretta, Antonino G. M. Marullo, Giacomo Frati

**Affiliations:** ^1^Department of Pathobiology and Medical Biotechnologies, University of Palermo, Palermo, Italy; ^2^Unit of Cardiac Surgery, Department of Surgery and Oncology, University of Palermo, Palermo, Italy; ^3^Department of Experimental Medicine and Surgery, University of Rome “Tor Vergata”, Rome, Italy; ^4^Department of General and Specialist Surgery, University of Rome “Sapienza”, 00161 Rome, Italy; ^5^Department of Medico-Surgical Sciences and Biotechnologies, Sapienza University of Rome, Latina, Italy; ^6^Department of Cardiovascular, Respiratory, Nephrological, Anesthesiological, and Geriatric Sciences, Sapienza University of Rome, Rome, Italy; ^7^IRCCS Neuromed, Pozzilli, Italy

## Abstract

Bicuspid valve disease is associated with the development of thoracic aortic aneurysm. The molecular mechanisms underlying this association still need to be clarified. Here, we evaluated the circulating levels of T and B lymphocyte subsets associated with the development of vascular diseases in patients with bicuspid aortic valve or tricuspid aortic valve with and without thoracic aortic aneurysm. We unveiled that the circulating levels of the MAIT, CD4+IL−17A+, and NKT T cell subsets were significantly reduced in bicuspid valve disease cases, when compared to tricuspid aortic valve cases in either the presence or the absence of thoracic aortic aneurysm. Among patients with tricuspid aortic valve, these cells were higher in those also affected by thoracic aortic aneurysm. Similar data were obtained by examining CD19+ B cells, naïve B cells (IgD+CD27−), memory unswitched B cells (IgD+CD27+), memory switched B cells (IgD−CD27+), and double-negative B cells (DN) (IgD−CD27−). These cells resulted to be lower in subjects with bicuspid valve disease with respect to patients with tricuspid aortic valve. In whole, our data indicate that patients with bicuspid valve disease show a quantitative reduction of T and B lymphocyte cell subsets. Future studies are encouraged to understand the molecular mechanisms underlying this observation and its pathophysiological significance.

## 1. Introduction

Bicuspid aortic valve disease (BAV) is a relatively frequent congenital disorder, affecting approximately 1.3% of the population worldwide with a male prevalence of 3 : 1 [1]. BAV is associated with an increased incidence of valvular and vascular diseases [1]. BAV is an important risk factor predisposing to the development of thoracic aortic aneurysm (TAA) [1, 2]. The molecular mechanisms underlying the association between BAV and aortic disease still need to be clarified [2].

In recent years, accumulating lines of evidence indicated that an increased inflammation of the aortic wall contributes to the development and progression of aortic aneurysm [3–5]. Inflammatory cytokines and an infiltrate of CD3+CD4+CD8+CD68+CD20+ cells have been demonstrated to significantly increase in human aneurysm specimens from patients with Marfan syndrome and familial and sporadic TAA [3–9]. B lymphocytes were also found to infiltrate the wall of aortic aneurysms significantly contributing to their expansion and progression [10]. Significant amounts of immune/inflammatory cells have been also detected by our group in aorta tissues from 24 BAV patients with TAA than control aortas, but with higher levels in individuals with tricuspid aortic valve (TAV) and affected by TAA [11]. However, their phenotypes and their possible differences were not assessed in our study [11]. Accordingly, some experimental studies in animal models have demonstrated that the attenuation of aortic immune/inflammation prevents or delays the progression of aortic aneurysm [3–6]. Pharmacological or genetic depletion of T helper lymphocytes and *γδ* T cells, a subset of T cells, was observed to reduce the progression of aortic aneurysms [12–15]. However, at the moment, no literature data do exist about the types of phenotypes of immune/inflammatory cells and their related number differences between patients with BAV and TAV, with and without concomitant TAA.

Therefore, in this study, we evaluated, for the first time, whether BAV subjects have typical signatures in peripheral blood immune cell levels and phenotypes, and particularly in T and B cell subsets, with respect to TAV subjects in the presence or absence of concomitant TAA. On the other hand, the presence of typical molecular, cellular, and genetic profiles in BAV patients with TAA in comparison to TAV with TAA continues to be evidenced in the literature, as amply stressed and demonstrated in our previous studies [9, 11, 16, 17].

## 2. Subjects and Methods

### 2.1. BAV and TAV Subjects

Our study included a total of 25 BAV subjects (19 males and 6 females; mean age: 56.7 ± 13.5 years) and 35 TAV subjects (23 males and 12 females, mean age: 66.4 ± 7.1 years). They were randomly selected from patients undergone to surgery replacement or routine care screening in the Unit of Cardiac Surgery (Department of Surgery and Oncology, University of Palermo), by using apposite exclusion criteria for arteriosclerosis or other cardiovascular diseases, connective tissue disorders, and inflammatory diseases (from infections to hematological, gastrointestinal, urogenital, pulmonary, neurological, and endocrinal inflammatory disorders and neoplasia included). They were enrolled from January 2015 to December 2016. Furthermore, we selected BAV and TAV individuals with or without TAA, as a complication, for evaluating appropriate controls for the same groups. In addition, they belonged to the same ethnic group, since their parents and grandparents were born in Western Sicily.

Elective or urgent surgical treatments (using Bentall-De Bono and Tirone David surgical techniques, whenever possible) with complementary tubular-ascending aorta resection were performed in both BAV and TAV patients with TAA after the evaluation of aortic transverse diameter sizes. The evaluation of aorta diameters was done preoperatively as well as in the operating theatre performed by an experienced physician by transesophageal echocardiography (*Philips Ie. 33*) before the institution of the cardiopulmonary bypass. The dimension of the aortic annulus, sinuses of Valsalva, proximal ascending aorta (above 2.5 cm of the sinotubular junction), and aortic arch are assessed and presented in Table 1.

Demographic and clinical data, including comorbidities, were obtained from patients' medical records (Table 1). In all BAV and TAV cases, hypertension was treated by using beta-blockers.

Blood samples were collected into EDTA-coated tubes from all individuals enrolled and at the moment of their admission in the Unit of Cardiac Surgery. They were transported to the laboratory and processed within 1 to 2 hours after the collection.

### 2.2. Ethical Study Approval

Our study was performed in accordance with ethical standards of the Helsinki Declaration of the World Medical Association; it received approval from local ethics committees (number APUNIP0094517), and all participants gave their informed consent. Data were encrypted in order to ensure the patient's privacy. All measurements were performed by physicians in a blind manner.

### 2.3. Antibody Panels and Multiparametric Flow Cytometry Analyses for Evaluating Circulating Levels and Phenotypes of T and B Cell Subsets

After separation from the whole blood in EDTA, 100 *μ*l of viable PBMC (*peripheral blood mononuclear cell*) was stained with different combinations of monoclonal antibodies. To characterize the phenotype of T and B cell subsets, extracellular labeling was performed with anti-CD8_FITC_, anti-CD161_PE_, anti-CD3_ECD_, anti-CD4_PC5.5_, anti-CD16_FITC_, anti-CD56_PE_, anti-IgD_FITC_, anti-CD27_PC5.5_, and anti-CD19_ECD_ (Beckman Coulter, Miami, FL). Living cells were gated within the side/forward scatter (SSC/FSC) lymphocyte gate. For intracellular staining, cells were permeabilized with Cytofix/Citoperm (BD Biosciences). Finally, the cells were stained with anti-IL-17A_FITC_ (MiltenyiBiotec), washed, and analyzed. All measurements were made with a CyAN ADP flow cytometer (Beckman Coulter, Miami, FL, USA) with the same instrument setting. At least 105 lymphocytes were acquired and analyzed using FlowJo (Tree Star) software. Leukocyte count and differential were determined with a routine hematology analyzer. The absolute counts of total lymphocytes were calculated by multiplying the relative size of the T and B cells and the absolute lymphocyte count.

### 2.4. Statistical Analysis

**Figure 1:**
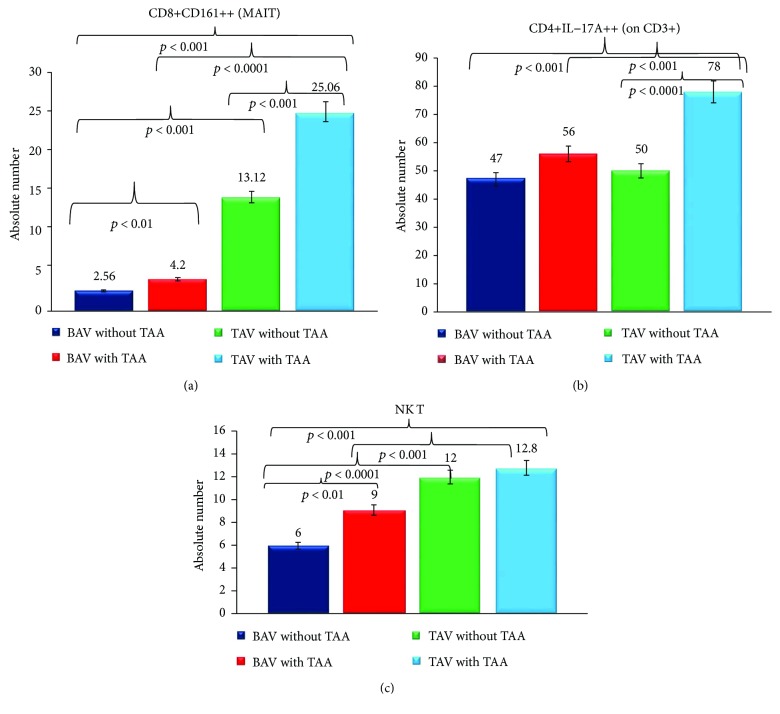
(a–c). Circulating levels of MAIT, CD4+IL−17A+, and NKT cells in the BAV and the TAV groups. Circulating MAIT and CD4+IL−17A+ levels were evaluated in patients with BAV and TAV with or without TAA. They were expressed as the absolute numbers (on CD3+). For the description of data, remand to Results.

**Figure 2:**
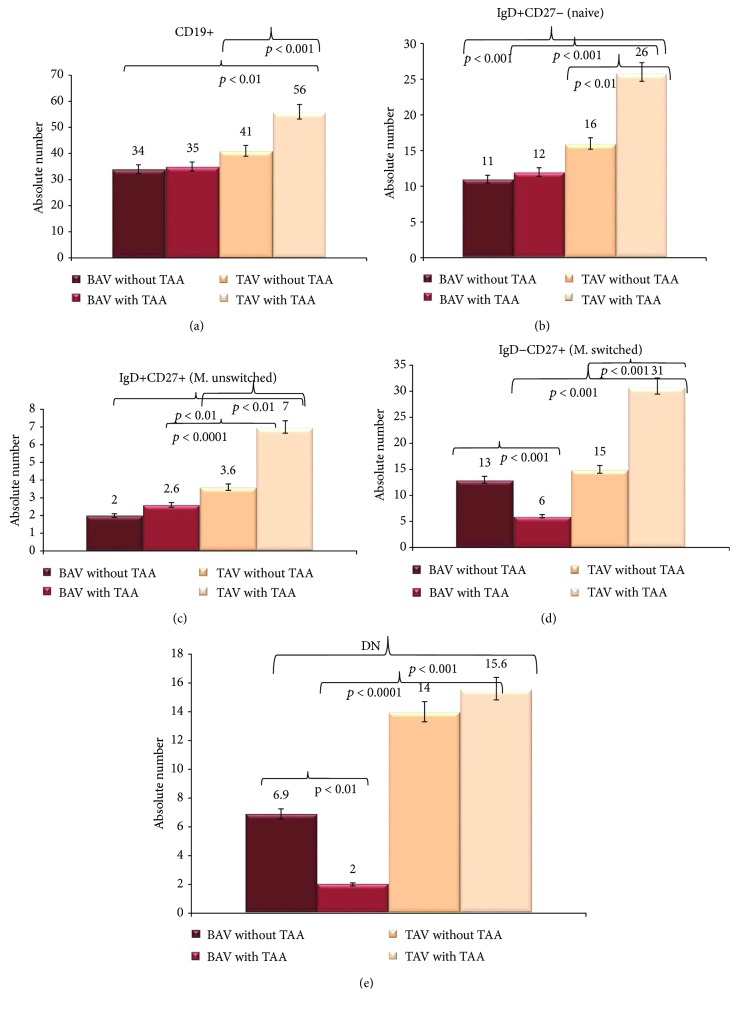
Circulating levels of B subsets in the BAV and the TAV groups. Circulating CD19+ B cells (a), naïve B IgD+CD27− cells (b), memory unswitched IgD+CD27+ B cells (c), memory switched B IgD−CD27+ cells (d), and double-negative B cells (e) were evaluated in patients with BAV and TAV with or without TAA. Cells were expressed as the absolute numbers. For the description of data, see Results.

As reported in Figures 1 and 2, the levels in the absolute number of MAIT, CD4+IL−17A+ and NKT T cells (for T compartment examined), and CD19+ B cells, naïve B cells (IgD+CD27−), memory unswitched B cells (IgD+CD27+), memory switched B cells (IgD−CD27+), and double-negative (DN) B cells (IgD−CD27−) (for B compartment evaluated) were expressed as the mean ± SD. Statistical analyses were performed using SPSS software version 20. Precisely, we used the analysis of variance (ANOVA) test (corrected by Bonferroni), for performing the comparisons among all groups. Unpaired *t*-test (Welch corrected) was utilised to analyze the data between two groups. Differences are considered significant when a *p* value < 0.05 was obtained by a comparison between the different groups.

## 3. Results

### 3.1. Patient and Control Characteristics

All BAV and TAV patient features are summarized in Table 1. A significant difference was observed in age between BAV and TAV patients. BAV patients were significantly younger than TAV cases (56.7 ± 13.5 versus 66.4 ± 7.1 years, resp., *p* < 0.0001). No significant differences were evidenced in the number of males and females between the two groups, as well as in the size of aorta dilatation between BAV and TAV cases. Among TAA risk factors, no significant differences were detected. We only observed in BAV cases a not significant prevalence of valvular complications compared to TAV cases.

### 3.2. Differences in the Circulating Levels of T Subsets in BAV and TAV Cases

We initially compared the circulating levels of Mucosal-associated invariant T (MAIT) cells in BAV versus TAV cases with and without TAA. MAIT cells represent a novel innate-like T cell subsets consisting of 1%–10% of T cells in the peripheral blood [18–20]. They mediate a pivotal role in immune-dysregulated diseases and other pathologies, like infections, inflammatory diseases, and others [18–20]. We observed that the mean blood levels of MAIT cells, expressed in absolute numbers, were significantly different between the BAV and the TAV groups, with and without TAA (see Figure 1(a)). Precisely, the comparisons among the four groups (see Figure 1(a)) demonstrated significant differences (*p* < 0.001, by the ANOVA test corrected by Bonferroni, in all comparisons effectuated), with the lowest blood levels in the BAV groups and more marked in the two TAV groups. A weak, but significant, the difference was assessed between the two BAV groups (*p* < 0.01, by the *t*-test corrected by Welch). Differently, between the two TAV groups, the mean blood levels of MAIT cells were significantly higher (*p* < 0.001, by the *t*-test corrected by Welch). Likewise, the comparisons of the mean levels of MAIT cells between BAV and TAV not complicated (*p* < 0.001, by the *t*-test corrected by Welch) and between BAV and TAV complicated (*p* < 0.0001, by the *t*-test corrected by Welch) were significantly different (see Figure 1(a)).

Similar results were observed regarding another T cell subset, namely, the CD4+IL−17A+, which has a recognized role in contributing to hypertension, vascular dysfunction, and damage [21–24]. Recent evidence suggests the participation of this T cell subset also in the development of aortic aneurysms [12, 22–24]. Circulating levels of CD4+IL−17A+, expressed in absolute numbers, were significantly different among the four groups (*p* < 0.001, by the ANOVA test corrected by Bonferroni, in all comparisons effectuated) (see Figure 1(b)). TAA was associated with significantly higher mean CD4+IL−17A+ levels in the TAV group. No difference between BAV and TAV subjects without TAA was assessed. In contrast, mean circulating CD4+IL−17A+ levels resulted to be significantly higher in TAV versus BAV subjects affected by TAA (*p* < 0.001, by the *t*-test corrected by Welch).

Furthermore, we also examined with regard to the T compartment the circulating levels of NKT cells, since they are involved in the genesis of atherosclerosis, coronary artery diseases, and aneurysms [25]. The comparisons of mean levels expressed in the absolute number of NKT cells among the four groups showed significant differences (*p* < 0.001, by the ANOVA test corrected by Bonferroni, in all comparisons effectuated) (see Figure 1(c)). In addition, the NKT population was markedly represented in TAV cases than BAV cases, in the presence or without TAA (*p* < 0.001 and *p* < 0.0001, by the *t*-test corrected by Welch, resp.; in particular, they were about the half in BAV versus TAV cases without TAA) (see Figure 1(c)). No differences were detected between the two TAV groups, while significantly higher mean levels of NKT cells were assessed in BAV TAA affected versus BAV subjects not affected (*p* < 0.01, by the *t*-test corrected by Welch) (see Figure 1(c)).

### 3.3. Variations in the Circulating Levels of B Cell Populations

As evidenced in Introduction, B cells contribute in the chronic immune/inflammatory pathophysiology of TAA [3–10]. Thus, we also evaluated the mean circulating levels expressed in the absolute number of CD19+ cells, naïve B cells (IgD+CD27−), memory unswitched B cells (IgD+CD27+), memory switched B cells (IgD−CD27+), and double-negative (DN) B cells (IgD−CD27−; i.e., exhausted memory cells) (see Figures 2(a)–2(e)) in the four groups. Regarding the CD19+ cells, significant differences were observed among the four groups (*p* < 0.01, by the ANOVA test corrected by Bonferroni, in all comparisons effectuated) (see Figure 2(a)). Furthermore, the comparisons detected significantly higher mean levels of the CD19+ cells in the TAV group with TAA versus the TAV group without TAA (*p* < 0.001, by the *t*-test corrected by Welch). No other differences were observed (see Figure 2(a)). Likewise, the mean blood levels expressed in the absolute number of the IgD+CD27− (naive) B subset were significantly different among the four groups (*p* < 0.001, by the ANOVA test corrected by Bonferroni, in all comparisons effectuated) (see Figure 2(b)). They resulted to be more marked in the BAV and the TAV groups with TAA, with higher mean values in TAV (*p* < 0.001, by the *t*-test corrected by Welch). Between the two BAV groups, no differences were detected, while higher mean values were assessed in TAV with TAA versus TAV without TAA (*p* < 0.01, by the ANOVA test corrected by Bonferroni, in all comparisons effectuated) (see Figure 2(b)).

Similarly, mean levels expressed in the absolute number of IgD+CD27+ (M. unswitched) B cells (see Figure 2(c)) were moderately different among the four groups (*p* < 0.01, by the ANOVA test corrected by Bonferroni, in all comparisons effectuated). The highest mean values were detected in TAV with TAA, which significantly differed from those in BAV affected by TAA (they were about 3 times in TAA) (*p* < 0.0001, by the *t*-test corrected by Welch) (see Figure 2(c)).

Interestingly, an opposite trend was observed in the BAV and the TAV groups with respect to IgD−CD27+ (M. switched) B subset levels in patients with and without TAA (see Figure 2(d)). The mean circulating levels of the IgD−CD27+ (M. switched) B subset expressed in the absolute number were more marked in BAV without TAA versus BAV with TAA (*p* < 0.001, by the *t*-test corrected by Welch; in BAV with TAA, the values were about the half of those of BAV with TAA). Significant differences were also detected between the two TAV groups (without versus with TAA, *p* < 0.001, by the *t*-test corrected by Welch) and between BAV and TAV affected by TAA (*p* < 0.001, by the *t*-test corrected by Welch) (see Figure 2(d)).

Finally, the mean circulating levels of DN B cells expressed in the absolute number (see Figure 2(e)) were significantly different among the four groups (*p* < 0.001, by the ANOVA test corrected by Bonferroni, in all comparisons effectuated). However, the lowest values were detected in BAV with TAA, which significantly differed with those of BAV without TAA with *p* < 0.01 (by the *t*-test corrected by Welch) and with those of TAV with TAA (*p* < 0.0001, by the *t*-test corrected by Welch). No differences were observed between the two TAA groups (see Figure 2(e)).

## 4. Discussion

Our study demonstrates that BAV is associated with a reduction in the circulating levels of some T and B lymphocyte subsets. BAV patients show a significant reduction in a number of all T and B subsets examined, with respect to TAV individuals, with a little trend in the increase (but not significant) in those with TAA (see Figures 1 and 2). Precisely, they have the lowest levels in MAIT and NKT cell subsets. The mean levels of the CD4+IL−17A+ cell subset were similar in the two BAV groups. But they also reflect the pattern of TAV patients without TAA, with the difference that likely in BAV patients they are not able to clonically expand and result as poorly functional, given the trend not in the increase with the TAA complication (see Figure 1(b)). The same results have been also obtained (see Figures 2(a)–2(d)) in all B subsets evaluated, with the exception of the DN B cell subset. These last showed an inverse trend with more pronounced levels in BAV individuals without TAA versus BAV individuals with TAA, but are like those in TAV without TAA (see Figure 2(e)). Differently, TAV individuals with or without TAA showed significant levels of all T and B subsets analyzed, with a significant trend in the increase in those affected by TAA (see Figures 1 and 2). Thus, the data obtained agree upon the results detected from our previous studies on BAV and TAA conditions [9, 11, 16, 17, 26]. Furthermore, they additionally confirm our previous suggestions about the presence in BAV individuals of unique cellular, molecular, and genetic mechanisms associated with TAA onset [11].

Previous studies' evidence indicated that lymphocytes play a significant role in the development and progression of aortic aneurysms. T lymphocyte infiltration is abundant in the aortic aneurysm wall in patients [3–9]. B lymphocytes were also found to infiltrate the wall of aortic aneurysms significantly contributing to their expansion and progression [10]. Significant amounts of T and B immune/inflammatory cells have been also detected by our group in aorta tissues from 24 BAV patients with TAA than control aortas, but with higher levels in individuals with tricuspid aortic valve (TAV) affected by TAA [11]. Animal studies showed that depletion of both T and B lymphocytes delays the progression of the disease [3–6]. Pharmacological or genetic depletion of T helper lymphocytes and *γδ* T cells, a subset of T cells, was, indeed, observed to reduce the progression of aortic aneurysms [12–15].

Surprisingly, even though the presence of BAV is associated with a higher incidence of TAA with respect to TAV, our data firstly indicate that this valvular defect is associated with a reduction in the circulating levels of some T and B lymphocyte subsets that usually take part to the chronic immune/inflammatory processes involved in the development of common cardiovascular diseases, such as TAA [6, 10, 18–25]. This data may suggest that the T and B lymphocyte activation is not likely the unique factor, which can contribute to the increased rate of the development of TAA in subjects with BAV. Probably, it might be hypothesized that the potential cause might be a close relationship between BAV itself condition and the compromised T and B compartment. Future studies are encouraged to test this hypothesis and to understand the molecular mechanisms underlying the reduced circulating lymphocyte levels in subjects with BAV. Previous studies have indicated that BAV patients more frequently carry mutations in the *NOTCH1 gene* [27–33]. Moreover, BAV condition presenting together with ascending aortic dilation has been also demonstrated to be significantly associated with other rare variants in the *NOTCH1 gene* [32, 33]. Notch signaling is an important regulator of inflammatory cell maturation and mobilization [27]. Therefore, it is possible that a defect of Notch signaling leads to a deregulation of inflammatory cells in patients with BAV.

## 5. Conclusions: Our Suggestions and Recommendations

The data obtained indicate that BAV subjects have significantly reduced levels in all T and B subsets examined. They lead us to propose some crucial suggestions, which might lead to new ways to research. They are summarized in Table 2. Firstly, this typical profile in T and B lymphocyte subsets would seem to suggest that BAV individuals may have an appropriate response to chronic tissue damage, and earlier than TAV individuals, that generally develop TAA disease in older ages [17]. Accordingly, this clinical situation seems to reflect what has been observed in older people, as demonstrated in previous studies from our and other groups [34–36]. In other words, the immune system in BAV cases would seem likely to appear as an “*old immune system*” with an altered specific clonotypic component and an increased innate/inflammatory compartment, which is consequently more easily vulnerable to internal and external stressors, frailty, disability, and disease [34–36]. In agreement with this suggestion, very marked levels of CD68+ monocyte cells have been observed by our and other groups in aorta specimens from BAV cases with TAA [3–9, 11]. This might likely justify their higher incidence of chronic immune/inflammatory vascular and aortic complications, such as TAA, at younger ages than TAV subjects.

Furthermore, this altered T and B immune profile in BAV patients, although evaluated only from a quantitative view point, may lead us likely to hypothesize the existence of a close relationship between BAV itself condition and the compromised T and B lymphocyte compartments. Indeed, it might speculate the existence in BAV individuals of alterations in pathways physiologically involved in two processes or in targets that were not exhaustively investigated in our study. In fact, aneurysm formation and progression are the outcomes of a complex process, in which more pathways, like Notch, Toll-like receptor-4, TGF-*β*, and so on, and their downstream components might play relevant roles, as elegantly stressed in a model proposed in our recent review published in a renowned journal [26]. This could also be the case of some proteases, whose function has been reported to be either protective or worsening depending on the aneurysm's location or with TGF-*β* signaling, recently demonstrated to be able to activate an autocrine IL-1*β* pathway acting as a signal recruiting innate immune cells in the adventitia through CCL2IL-1*β* [37].

In our case, a pathway that mediates these functions is the Notch pathway, characterized by 4 type transmembrane receptors (Notch1–Notch4) in human. In our specific case, our attention is focused on Notch 1. It has been shown to have pleiotropic effects: stem/progenitor cell fate; regulation of the life cycle of adult cells and regulation of multiple steps of T and B cell development in both central and peripheral lymphoid organs; and the development of cardiovascular system and so on, as amply described in our recent review [27]. Furthermore, BAV has been significantly associated with rare but highly penetrant exonal mutations in the *NOTCH1* gene [27–33], and BAV presenting together with ascending aortic dilation has been also demonstrated to be significantly associated with other rare variants in the *NOTCH1 gene* [32, 33]. Accordingly, an impairment of Notch signaling has been also shown to be involved in the development and progression of aortic aneurysm, as elegantly summarized in 2017 by Yassine and co-workers [38] and recently demonstrated by our group in a recent study (data not shown).

Certainly, additional and larger studies are mandatory to confirm these promising findings as well as our suppositions. The replication of our results from a very large sample size might give the possibility of detecting potential molecular, cellular, and genetic biomarkers to be translated in the daily clinical practice. In turn, they might consent to identify BAV subjects at high risk to develop TAA and to provide an appropriate guidance about their treatments, which might be different from those for TAV subjects with TAA. This could consent us to suggest specific clinical recommendations on the surgical approaches to apply in the case of BAV patients with TAA. The selection of drug therapies and more suitable surgical procedures with or without composite aortic root replacement represents the major object of current cardiovascular studies. Based on our previous and current data, we suggest to go beyond the values of the aorta's diameter and growth rate as the only parameters for the surgical recommendation [39]. The surgical strategy would also consider not only the clinical features but also the molecular, cellular, and genetic profiles of everyone and particularly in the case of BAV condition. It would be interesting to initiate discussion about personalized therapeutic and surgical recommendations, particularly in the case of BAV condition.

## Figures and Tables

**Table 1:** Demographic and clinical characteristics, comorbidity conditions, and complications of 25 BAV and 35 TAV subjects with or without TAA.

Variables	BAV	TAV
	*N* = 25	*N* = 35
*Demographic characteristics*		
Age, mean (SD)	56.7 (13.5)	66.4 (7.1)
Male sex, number (%)	19 (76)	23 (66)
Female sex, number (%)	6 (24)	12 (34)
Body mass index, mean (SD)	26 (4.8)	26.3 (3.2)
*Size and location of TAA*		
Subjects affected (%)	12 (48)	17 (48)
Size (mm), mean (SD)	53.3 (7.4)	50.3 (6.9)
Location, number (%):		
Tubular ascending aorta	12 (100)	17 (100)
*Comorbidity conditions, number (%)*		
CVD family history	7 (28)	5 (7.1)
Smoking	6 (24)	7 (20)
Hypertension	18 (72)	25 (71)
Dyslipidemia	3 (12)	5 (14)
Diabetes mellitus	0 (0)	0 (0)
Renal failure	0 (0)	1 (2)
Dissection	0 (0)	0 (0)
*Aortic valve pathology, number (%)*		
Normal	0 (0)	27 (77)
Prolapse	3 (12)	1 (2)
Vascular calcium fibrosis	7 (28)	7 (20)
*Atherosclerosis coronary syndrome, number (%)*	0 (0)	0 (0)

**Table 2:** Our findings and suggestions.

Findings	Suggestions
BAV subjects showed the lowest levels in MAIT and NKT cell subsets for T compartment examined (see all Figures 1(a)–1(c). Despite this, they surprisingly had mean levels of CD4+IL−17A+ cell subset, which were like those of TAV without TAA. While for the B compartment, the BAV individuals, independent to TAA disease, showed low and similar levels in the two groups (see Figures 2(a)–2(d)) in all B subsets evaluated, with the exception of the DN B cell subset. These last showed an inverse trend with more pronounced levels in BAV without TAA versus BAV individuals with TAA, but are like those in TAV without TAA (see Figure 2(e)).	(1) They would suggest that BAV individuals may have unaltered response to chronic tissue damage and, earlier than TAV individuals, that generally develop TAA disease in older ages [17]. (2) Although, they show a CD4+IL−17A+ cell subset that would seem to be not compromised, but not able probably to clonically expand, or poorly functional. Accordingly, the significantly reduced numbers of T and B lymphocyte subsets from BAV individuals would be likely similar to those observed in older people, as demonstrated in previous studies by our and other groups [33–35]. (3) The immune system in BAV cases would seem likely to appear as an “*old immune system*” with an altered specific clonotypic component and an increased innate/inflammatory compartment, which is consequently more easily vulnerable to internal and external stressors, frailty, disability, and disease [27–29]. In agreement with this suggestion, very marked levels of CD68+ monocyte cells have been observed by our and other groups in aorta specimens from BAV cases with TAA [3–9, 11]. (4) This might likely justify their higher incidence of chronic immune/inflammatory vascular and aortic complications, such as TAA, at younger ages than TAV subjects. (5) A close relationship between BAV itself condition and the compromised T and B lymphocyte compartments would seem to be the cause of this altered T and B profile. Here, we hypothesize that defects in the Notch signaling pathway may be the close link between the deregulated T and B response and the BAV itself condition.

TAV individuals with or without TAA showed very significant levels of all T and B subsets analyzed, with a significant trend in augment in those affected by TAA (see Figures 1 and 2). The data obtained agree the results detected from our previous studies on BAV and TAA conditions.	(1) In BAV individuals, unique cellular, molecular, and genetic mechanisms are associated with TAA onset [11].
